# Defective Interfering Viral Particles in Acute Dengue Infections

**DOI:** 10.1371/journal.pone.0019447

**Published:** 2011-04-29

**Authors:** Dongsheng Li, William B. Lott, Kym Lowry, Anita Jones, Hlaing Myat Thu, John Aaskov

**Affiliations:** 1 Institute of Health and Biomedical Innovation, Queensland University of Technology, Brisbane, Queensland, Australia; 2 Virology Research Division, Department of Medical Research, Yangon, Myanmar; Institut Pasteur, France

## Abstract

While much of the genetic variation in RNA viruses arises because of the error-prone nature of their RNA-dependent RNA polymerases, much larger changes may occur as a result of recombination. An extreme example of genetic change is found in defective interfering (DI) viral particles, where large sections of the genome of a parental virus have been deleted and the residual sub-genome fragment is replicated by complementation by co-infecting functional viruses. While most reports of DI particles have referred to studies *in vitro*, there is some evidence for the presence of DI particles in chronic viral infections *in vivo*. In this study, short fragments of dengue virus (DENV) RNA containing only key regulatory elements at the 3′ and 5′ ends of the genome were recovered from the sera of patients infected with any of the four DENV serotypes. Identical RNA fragments were detected in the supernatant from cultures of *Aedes* mosquito cells that were infected by the addition of sera from dengue patients, suggesting that the sub-genomic RNA might be transmitted between human and mosquito hosts in defective interfering (DI) viral particles. *In vitro* transcribed sub-genomic RNA corresponding to that detected *in vivo* could be packaged in virus like particles in the presence of wild type virus and transmitted for at least three passages in cell culture. DENV preparations enriched for these putative DI particles reduced the yield of wild type dengue virus following co-infections of C6–36 cells. This is the first report of DI particles in an acute arboviral infection in nature. The internal genomic deletions described here are the most extensive defects observed in DENV and may be part of a much broader disease attenuating process that is mediated by defective viruses.

## Introduction

Inhibition of viral replication by what would become known as defective interfering (DI) particles was first described more than 60 years ago with influenza virus [1]. These non-infectious viral particles [2] were believed, at the time, to have lost an entire RNA genome segment [3], requiring complementation by a non-defective homologous virus for reproduction. DI particles, which now have been observed in a wide variety of viral populations, arise as a sub-population during infection by wild type virus and are defined by the following criteria [4]. A DI particle must (1) contain normal parental viral protein; (2) contain part of the parental viral genome; (3) require a parental helper virus to reproduce; and (4) interfere specifically with the intracellular replication of the parental virus. Populations of viruses containing “incomplete” virions have been found to be less virulent for experimental animals than the population from which they were derived [5].

DI particles have been observed most commonly *in vitro* during serial passages of viruses at high multiplicities of infection, and often have been invoked to explain the conversion of acute *in vitro* infections to persistent ones [6]–[11]. A role for DI particles in maintaining persistent infections *in vivo* also has been proposed e.g. measles virus and sub-acute sclerosing panencephalitis [12] and chronic hepatitis C infections [13], [14]. Whether DI particles are present in acute viral infections that do not lead to chronic infections in their natural hosts and the role of DI particles in acute infections are less well documented [15].

Dengue viruses (DENV) are arboviruses in the family *Flaviviridae* and are important human pathogens responsible for disease states described formerly as dengue fever, dengue haemorrhagic fever and dengue shock syndrome [16]. DENV are transmitted to humans by *Aedes* mosquitoes, principally by *Aedes aegypti*. The four closely related DENV serotypes are antigenically distinct and often co-circulate in tropical regions where this disease is endemic. The nucleotide sequences of the single-stranded positive-sense RNA genomes of DENV are very diverse, and most viral genomes recovered from either of the natural hosts contain defects (e.g. intra-genic stop codons, nucleotide insertions or deletions) that would render them non-infectious [17], [18]. Some of these defects appear to be maintained during natural cycles of transmission, presumably due to complementation by fully functional genomes [18]. We have suggested that these defective genomes impose a fitness burden on the DENV populations in which they are found that may result in attenuation of disease severity, allowing greater mobility of infected human hosts and therefore greater dissemination of virus [18], [19].

In this study, we used a modified 3′ RACE protocol to determine whether sub-populations of DENV in patients contained genomes with large internal deletions similar to those found in DI particles in other viral populations [20], [21] and whether viruses with these defective genomes could replicate in the presence of fully functional DENV.

## Results

### DENV genomes containing large internal deletions were detected in sera from dengue patients and in the corresponding isolates

**Figure 1:**
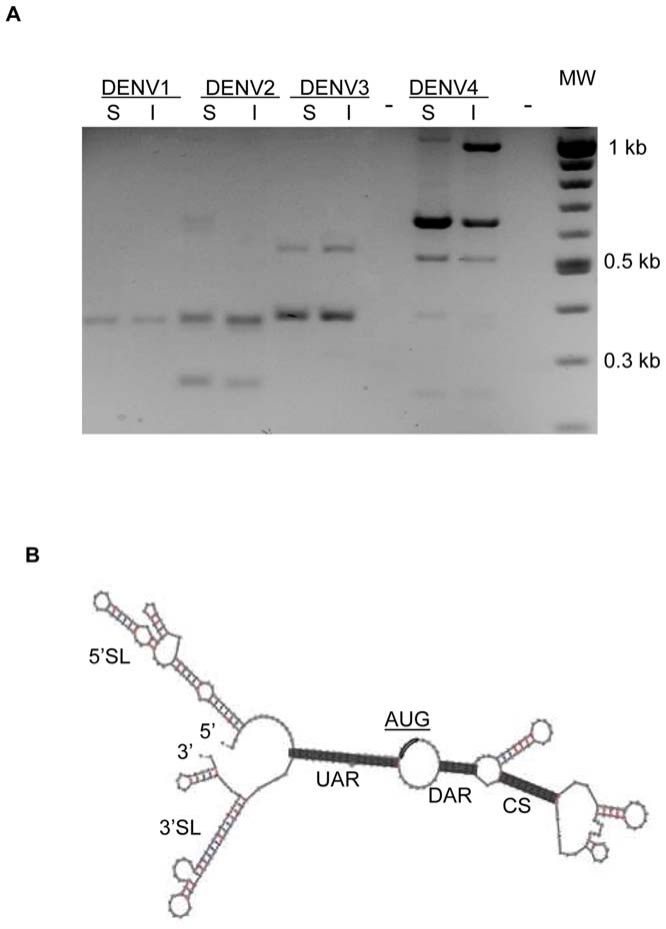
cDNA generated by 3′ RACE using primers corresponding to the 3′ and 5′ ends of the DENV genome. (A) Employing RNA extracted from sera [S] from dengue patients or from the corresponding isolates [I] (B) Predicted secondary structure of the 290 nt sub-genomic DENV 2 RNA showing elements referred to in the text. The AUG codon at the 5′ end of the open reading frame is underlined.

RNA extracted from sera from dengue patients and from the corresponding DENV isolates were subjected to modified 3′ RACE using oligonucleotide primers corresponding to the 3′ and 5′ ends of the DENV genome. PCR extension times of 2 minutes yielded cDNA fragments of approximately 1 kb or less (Figure 1A). PCR extension times of 6 minutes did not produce larger cDNA fragments. While cDNAs derived from sera and from their corresponding isolates usually were of the same size, the number of bands and their apparent molecular weights appeared to be characteristic of the DENV serotype. RNA recovered from sera from 4/8 DENV 1, 2/2 of DENV 2, 2/3 of DENV 3 and 9/9 DENV 4 patients gave rise to low molecular weight bands of cDNA similar to those shown in Figure 1A (Table S 1).

Low molecular weight cDNA fragments corresponding to those in Figure 1A were cloned and sequenced. With the exception of the 550 bp product generated from DENV 3 (discussed below), cDNA bands from all four DENV serotypes corresponded to DENV genomes with large internal deletions. The sequences corresponded to those of the 5′ UTR (including the 5′ upstream AUG region [UAR]), the motif downstream of the AUG region (DAR), some intervening sequence and the 3′ end of the 3′ UTR including the 3′ conserved sequence (CS) and the 3′ UAR (Table 1). The interface between the 5′ and 3′ elements included a variable-length “overlap” sequence found in the 5′ and 3′ regions of genomes of the corresponding serotypes (Table 1). The predicted secondary structure of the 290 nt sub-genomic DENV 2 RNA with all the elements referred to above is shown in Figure 1B. The AUG codon at the 5′ end of the open reading frame is underlined. The predicted secondary structures of all sub-genomic RNA in Table 1 resembled that in Figure 1 B, varying only in the predicted structure of the RNA between the 5′ and 3′ CS (data not shown).

**Table 1:** Nucleotide sequences at the putative junction between 5′ and 3′ regions of DENV genomes in sub-genomic RNA.

Source of RNA	size of cDNA(bp)	5′ sequence†	3′ sequence†	Nucleotide sequence at overlap region ‡
D1-67380 serum	370	nt 1-257	nt 10620-10735	5′ **ATAGCATTCCTAA**GATTTCTAGC ^267 10610^ ACCCCCGGCATAA **CAATAAACAG** 3′
D1-67380 isolate	370	nt 1-257	nt 10620-10735	5′ **ATAGCATTCCTAA**GATTTCTAGC ^267 10610^ ACCCCCGGCATAA **CAATAAACAG** 3′
D2-63662 serum	290	nt 1-179	nt 10607-10723	5′**GTCGACTGTG-AACA**GGCCCTGG ^187 10595^ AGACCCCCCCGAAACA **AAAACAGCAT**3′
	400	nt 1-213	nt 10526-10723	5′ **CTCACTTGGAAT-GC**TGCAAGGGAC ^218 10512^ CTCCCTTACAAATCGC **AGCAACAATG**3′
D2-63662 isolate	290	nt 1-179	nt 10607-10723	5′**GTCGACTGTG-AACA**GGCCCTGG ^187 10595^ AGACCCCCCCGAAACA **AAAACAGCAT**3′
	400	nt 1-213	nt 10526-10723	5′ **CTCACTTGGAAT-GC**TGCAAGGGAC ^218 10512^ CTCCCTTACAAATCGC **AGCAACAATG**3′
D3-66178 serum	400	nt 1-189	nt 10489-10684	5′**GTTGGCGAAGAG**ATTCTCAAAAA ^199 10480^ CAGACTAGCGG **TTAGAGGAGAC**3′
D3-66178 isolate	400	nt 1-189	nt 10489-10707	5′**GTTGGCGAAGAG**ATTCTCAAAAA ^199 10480^ CAGACTAGCGG **TTAGAGGAGAC**3′
D4-61673 serum	510	nt 1-404	nt 10534-10650	5′**GGAAAAGGTCAACA**TAACATTGCT ^414 10524^ GAGACCCCCCCAACA **CAAAAACAG** 3′
	690	nt 1-336	nt 10284-10650	5′ **ACAAGGCCATAAAGA**TACTAACTGG ^336 10274^ AAAAAACACCAAAGA **GGCTATTGAA** 3′
	830	nt 1-293	nt 10094-10650	5′**ACCAACAGCAGGGATT**CTGAAAAGAT ^301 9995^ GGAAAAGAGAGGATT **TGTGGTGTGG** 3′
D4-61673 isolate	510	nt 1-404	nt 10534-10650	5′**GGAAAAGGTCAACA**TAACATTGCT ^414 10524^ GAGACCCCCCCAACA **CAAAAACAG** 3′
	690	nt 1-336	nt 10284-10650	5′ **ACAAGGCCATAAAGA**TACTAACTGG ^336 10274^ AAAAAACACCAAAGA **GGCTATTGAA** 3′
	1030	nt 1-335	nt 9620-9712nt 10037-10650	5′**ACAAGGCCATAAAG**TACTAACTGG ^344 9610^ ACGACATGGGAAAG **GTGAGGAAAG** 3′ 5′**ACTTCCACAAGAT**CTTTATGAAG ^9723 10027^ ACCCTAATATGAT **TGACAAAACTC** 3′
DENV2 derived from infectious clone	391	nt1-190	10523-10724	5′ **GCTGACAAAGA**GATTCTCACT ^200 10513^ CCCCTCCCTTA **CAAATCGCAGCAA** 3′
DENV2 derived from infectious clone	362	nt1-177	10536-10724	5′ **TGTCGACTGTACAA**CAGCTGAC ^177 10528^ TCGCAGCAACAA **TGGGGGCCCAA** 3′
DENV2 derived from infectious clone	288	nt1-173	10609-10724	5′**GTCGACTGTACAACA**GCTGGACA ^187 10599^ GACCCCCCCAAAACA **AAAAACAGC**3′

The reference sequences for DENV-1, 2, 3 and 4 are AY726554.1, AF169688.1, FN429913.1 and AY618990.1 respectively. The accession number of the sequence of the DENV 2 infectious clone is AF038403.1.

‡ Sequence of the cDNA derived from subgenomic RNA is shown in bold. The sequence in plain text is from the corresponding reference sequence, and the underlined sequence is the overlap.

No non-DENV sequences were detected in these short fragments of RNA, and there were no nucleotide insertions, repeats or other aberrations. We were unable to recover sufficient of the 1100 bp cDNA generated from the serum of a DENV 4 patient (Figure 1 A) to be able to clone and sequence it. The 550 bp fragment derived from the serum of a patient with a DENV 3 infection and the corresponding isolate corresponded to the 5′ region of the DENV 3 genome attached to a 3′ primer used for PCR.

**Figure 2:**
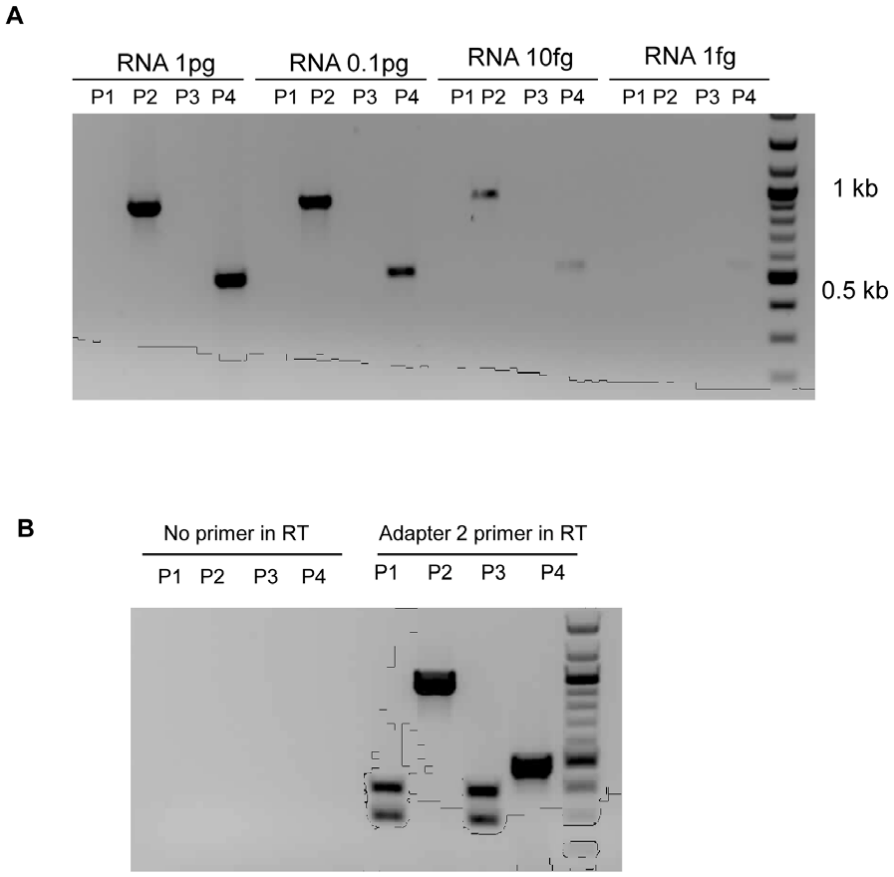
Effect of the concentration of DENV 2 RNA template and of the primers used for reverse transcription in 3′RACE on the generation of cDNA. (A) Analysis of cDNA produced following 3′ RACE employing ten-fold dilutions of RNA transcribed from a DENV 2 infectious clone as template and a variety of primer combinations [P1, D2-5′UTR-F and D2-3′UTR; P2, D2-9678-F and adaptor 2; P3, D2-5′UTR-F and adaptor 2; P4, D2-8375-F and D2-8885-R]. (B) cDNA generated following 3′ RACE RT-PCR with RNA from a DENV 2 isolate as template and the RT step performed with or without an oligonucleotide primer and a variety of primer combinations in PCR. [P1, D2-5′UTR-F and adaptor 2; P2, D2-9678-F and adaptor 2; P3, D2-5′UTR-F and D2-3′UTR-R; P4, D2-8375-F and D2-8885-R].

To determine whether all of these small cDNA fragments could have been artefacts generated from full length DENV genomes during RT-PCR, genome length DENV 2 RNA was transcribed from an infectious clone (pWSK601) and a ten thousand-fold range of dilutions of the RNA were utilised as templates in 3′RACE. No small cDNA fragments were generated when using primers corresponding to the 3′ and 5′ ends of DENV genome (P1), or with primers corresponding to 5′ end of DENV genome and complimentary to the nucleotide sequence of the adapter used for 3′ RACE (P3) (Figure 2 A). Thus, the primers that gave rise to small cDNA fragments when using RNA from the sera of dengue patients and from the corresponding isolates (Figure 1 A) failed to produce small cDNA fragments from *in vitro* transcribed full length DENV genomic RNA. In the positive control experiments, products of the predicted size were produced when using primers corresponding to an intragenic region of DENV 2 NS5 (P4) or one of the NS5 primers and a primer complimentary to the adaptor used for 3′ RACE (P2).

As an additional control for “self priming” during PCR [22], DENV2 isolate 62663 first was treated with 10 µg/ml of RNAse A for 10 min at 37°C to eliminate any unpackaged RNA or other RNA contaminant. This treatment was sufficient to destroy at least 50 ng sub-genomic RNA (data not shown). The RT step of the subsequent 3′ RACE was performed with or without a primer. No product was generated using any combination of primers in PCR if the RT reaction was performed without a primer (Figure 2 B). In contrast, two bands of cDNA, the same size as those generated previously from DENV 2 63662 (Figure 1 A), were observed when a primer complimentary to the 3′adaptor was employed in the RT reaction.

### Detection of sub-genomic RNA in northern blots

C6-36 cells were infected with DENV 2 isolate (61452) which, in addition to a full length genome, contained 290 nt sub-genomic RNA that could be detected by RT PCR (Table S1). Total RNA from infected cells and RNA from virions in the culture supernatant were extracted 5 and 8 days post-infection. Northern blot analyses were performed using DIG-labelled negative-sense RNA probes corresponding to the DENV2 5′1-206 nt (5′-probe) or the 3′10526-10723 nt (3′-probe). Genomic length DENV 2 RNA transcribed from infectious clone pWSK601and a 290 nt sub-genomic fragment of DENV 2 RNA transcribed from a plasmid containing the corresponding cDNA (Table 1) were recognised by both the 5′ and 3′ probe (Figure S1). Neither probe recognised RNA from uninfected cells. While it was possible to detect sub-genomic (∼290 bp) DENV RNA in infected cells and in supernatant from the infected cultures, the 3′ probe hybridised only weakly with genome length RNA from supernatant, perhaps reflecting the limited sensitivity of the assay (approximately 10 pg RNA),

### Negative strand sub-genomic RNA in DENV-infected cells

A strand-specific RT-PCR was employed to detect negative strand sub-genomic RNA in putative double strand replicative intermediates in DENV 2 (61452) infected C6-36 cells. The assay allowed approximately 1,000∼10,000 fold discrimination between positive and negative strand RNA using template concentrations between 0.001 and 1 pg of DENV 2 sub-genomic positive and negative strand RNA (Table 2). Negative strand RNA corresponding to the 290 nt fragment was detected in cells 2, 5 and 8 days post-infection as was corresponding positive strand RNA. The differences between copy numbers for negative and positive strand RNA were too small (see discriminatory values above) for the values for negative strand RNA to have been an artefact.

**Table 2:** Number of copies of negative and positive strand sub-genomic RNA (log_10_) in DENV 2 (61452) infected C6-36 cells.

	Sense primer in RT		Anti-sense primer in RT	
Control RNA	Neg. strand RNA	Pos. Strand RNA	Neg. strand RNA	Pos. Strand RNA
1 pg RNA	6.7	2.9	3.0	6.8
0.1 pg RNA	5.7	2.0	2.3	5.7
0.01 pg RNA	4.7	1.4	1.9	4.7
0.001 pg RNA	3.7	0.8	1.0	3.8
Cellular RNA				
Day 2 post infection	4.5			5.2
Day 5 post infection	4.3			5.0
Day 8 post infection	4.1			5.9

### DENV particles containing sub-genomic RNA could be generated from WT DENV-2 *in vitro*

**Figure 3:**
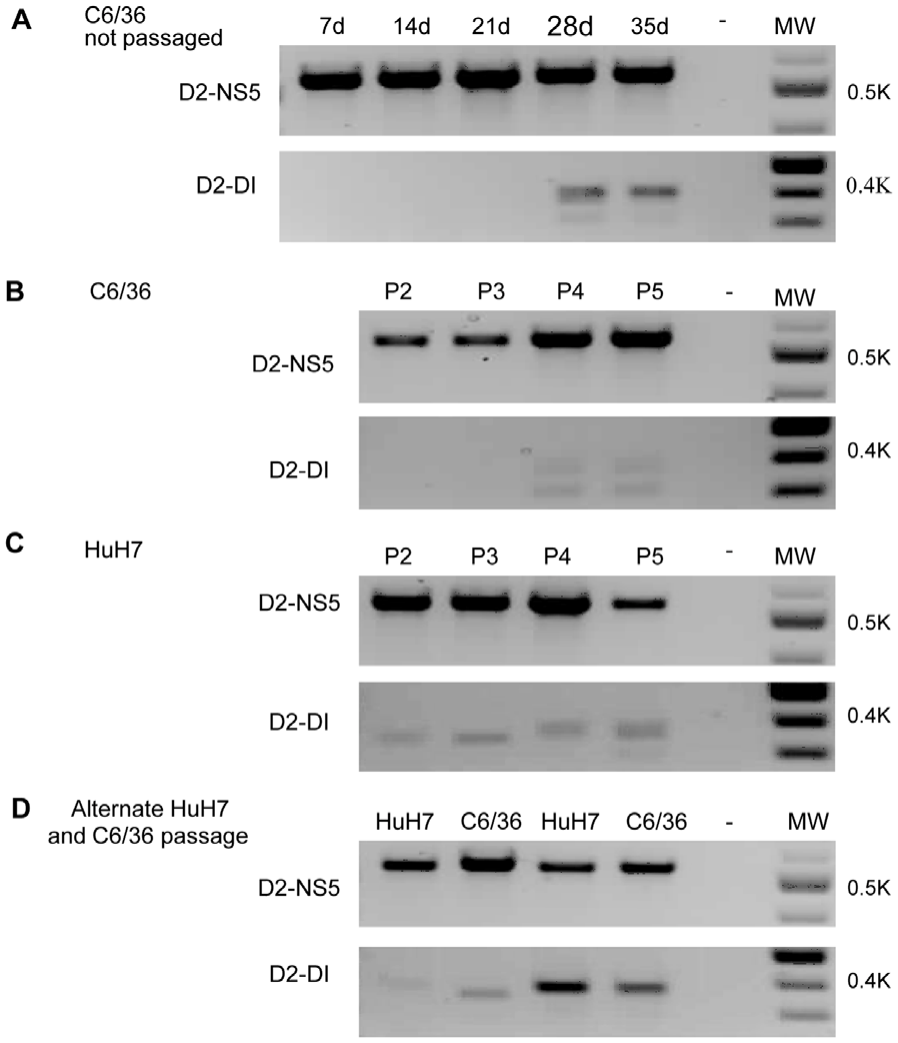
Generation of sub-genomic DENV 2 RNA during passage of wild type virus. DENV 2 derived from an infectious clone was passaged in C6-36 cells, in HuH7 cells or alternatively in both cell types. (A) C6-36 cells infected with infectious clone-derived virus and culture supernatant sampled at the times shown. (B) Virus passaged to uninfected cultures of C6-36 cells every 7 days. (C) Virus recovered from cultures of C6-36 cells after 7 days and thereafter passaged to uninfected cultures of HuH7 cells every 7 days. (D) Virus recovered from cultures of C6-36 cells after 7 days and thereafter passaged, alternately, into uninfected cultures of HuH7 cells or C6-36 cells every 7 days. (P2, passage 2; P3 passage 3 etc).

To determine if DENV particles containing sub-genomic RNA could be generated *in vitro* from a virus stock that contained only full length genomes, C6/36 mosquito cells were transfected with RNA that had been transcribed from a DENV-2 infectious clone (pWSK601). This culture was maintained by replacing the culture medium and assaying for sub-genomic DENV RNA every seven days. Sub-genomic DENV RNA was not detected in the supernatant from these cultures until 28 days post-infection (Figure 3 A). To simulate successive infection cycles, culture supernatant recovered 7 days after transfection of the C6/36 cells was passaged at intervals of 7 days in C6/36 mosquito cells, HuH7 human liver cells or in alternating passages of C6/36 and HuH7 cells. While sub-genomic DENV RNA was not detected in the culture supernatant until after four passages (28 days) in C6/36 mosquito cells (Figure 3 B), it could be detected after only one passage (7 days) in HuH7 human cells (Figures 3 C and D). The three small cDNAs derived by 3′ RACE with RNA from the supernatants of these cultures (288, 362, 391 bp) were cloned and sequenced. They corresponded to the 5′ and 3′ ends of the genome of the DENV 2 infectious clone and differed only at the point at which these elements had combined (Table 1, DENV 2 derived from infectious clone). The overlap between the 3′ and 5′ ends of the 288 nt sub-genomic RNA generated *in vitro* from genome length RNA derived from the infectious clone occurred in the same region as the overlap between the 3′ and 5′ ends of the 290 nt sub-genomic RNA from the serum of a dengue patient. The nt sequence (ACA) at the junction of the 288/290 sub-genomic RNAs was the same as that at the junction of the 3′ and 5′ ends of the 362 nt sub-genomic RNA generated during passage of the infectious clone-derived DENV 2 (Table 1).

### WT DENV complemented defective DENV RNA in cell culture

**Figure 4:**
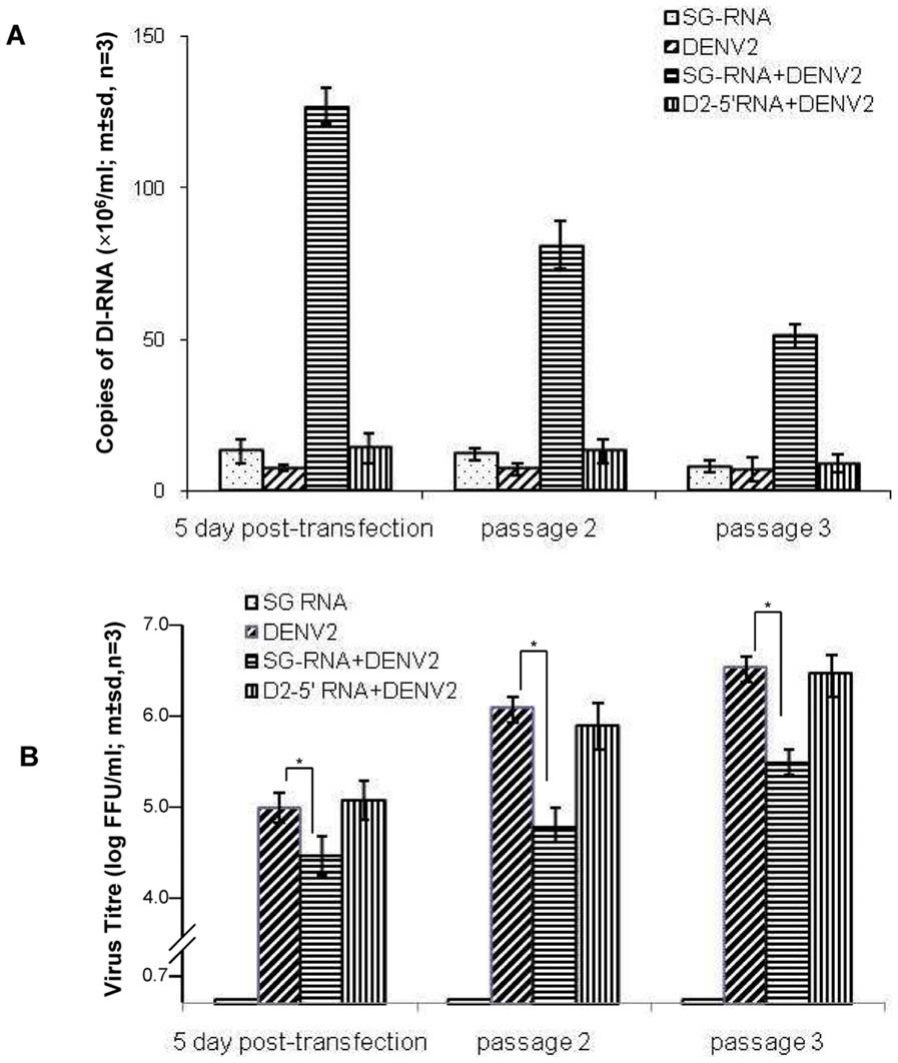
Replication of sub-genomic RNA in the presence of wild type virus. (A) Yield of sub-genomic (SG) DENV 2 RNA in cultures infected with infectious clone-derived DENV 2 and mock transfected (no RNA) or transfected with sub-genomic DENV 2 RNA corresponding to that detected in serum from dengue patients or with a control RNA preparation (D2-5′RNA, RNA corresponding to the 5′ 370 nt of the DENV 1 genome). (B) Yield of infectious virus from the cultures in (A). An asterix indicates the titres were significantly different (p<0.05, student t-test).

To determine if wild type DENV could complement sub-genomic DENV RNA *in trans* to yield particles containing sub-genomic RNA, C6/36 cells were infected with DENV 2 derived from infectious clone cDNA (pWSK601, no detectable defective RNA) and then transfected 24 h later with a 290 nt RNA that was transcribed *in vitro* from cloned cDNA (pUC-D2-DI) corresponding to the 290 nt sub-genomic DENV 2 RNA (Table 1). DENV 2 infected C6/36 cells also were mock transfected or transfected with RNA corresponding to the 5′-terminal 370 nt of the DENV 2 genome. Uninfected C6/36 cells were transfected with the 290 nt DENV RNA as an additional control. At each passage, culture supernatant was treated with mixture of RNase A and RNase T1, to digest any unprotected RNA contaminant, prior to RNA extraction. DENV 2 290 nt sub-genomic RNA was detected in the culture supernatant from DENV 2 infected C6/36 cells 5 days after transfection with DENV 2 290 nt RNA but not in culture supernatant from un-infected cells (not shown), or from DENV 2 infected C6-36 cells that had been mock transfected or transfected with the RNA corresponding to the 5′ 370 nt of the DENV 2 genome (Figure 4 A). Supernatant from these cultures were passaged a further two times in C6/36 cells at intervals of 7 days. Decreasing numbers of copies of DENV 2 290 nt sub-genomic RNA were detected with each passage (Figure 4 A). While there were no differences between the titres of DENV in the supernatant of cultures infected with DENV 2 and mock transfected or infected with DENV 2 and transfected with 5′ DENV RNA (Figure 4 B), the titres of DENV 2 in supernatant from cell cultures infected with DENV2 and transfected with the 290 nt RNA were significantly lower (p≤0.05, student t-test) than the titres of DENV2 in cultures of C6-36 cells infected with DENV 2 and mock transfected or transfected with 5′ DENV RNA. No infectious DENV was detected in the supernatant from cultures of uninfected C6-36 cells transfected with sub-genomic RNA.

**Figure 5 pone-0019447-g005:**
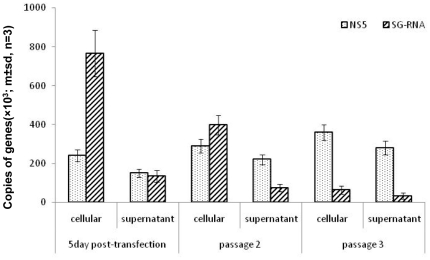
Comparison of the ratios of sub-genomic to genome length DENV RNA in C6-36 cells infected with infectious clone-derived DENV 2 and transfected with sub-genomic DENV 2 RNA and in the accompanying culture supernate (from **Figure 4**). One hundred ng of RNA from infected cells and RNA from the equivalent of 1 ul of culture supernate were used in the quantitative RT PCR reactions.

There was a significant excess of sub-genomic DENV RNA over full length DENV RNA in the cells infected with DENV 2 and transfected with sub-genomic RNA (p≤0.01, student t-test) (Figure 5). However, in the accompanying culture supernatant, the ratio of full-length to sub-genomic RNA was almost 1∶1. By passage 3, the ratios of full-length to sub-genomic DENV RNA in cells and in the corresponding culture supernatant were almost the same and there was a significant excess of full length over sub-genomic RNA (p≤0.01, student t-test).

### Populations of DENV enriched for particles containing sub-genomic RNA inhibited replication of WT DENV *in vitro*

**Figure 6:**
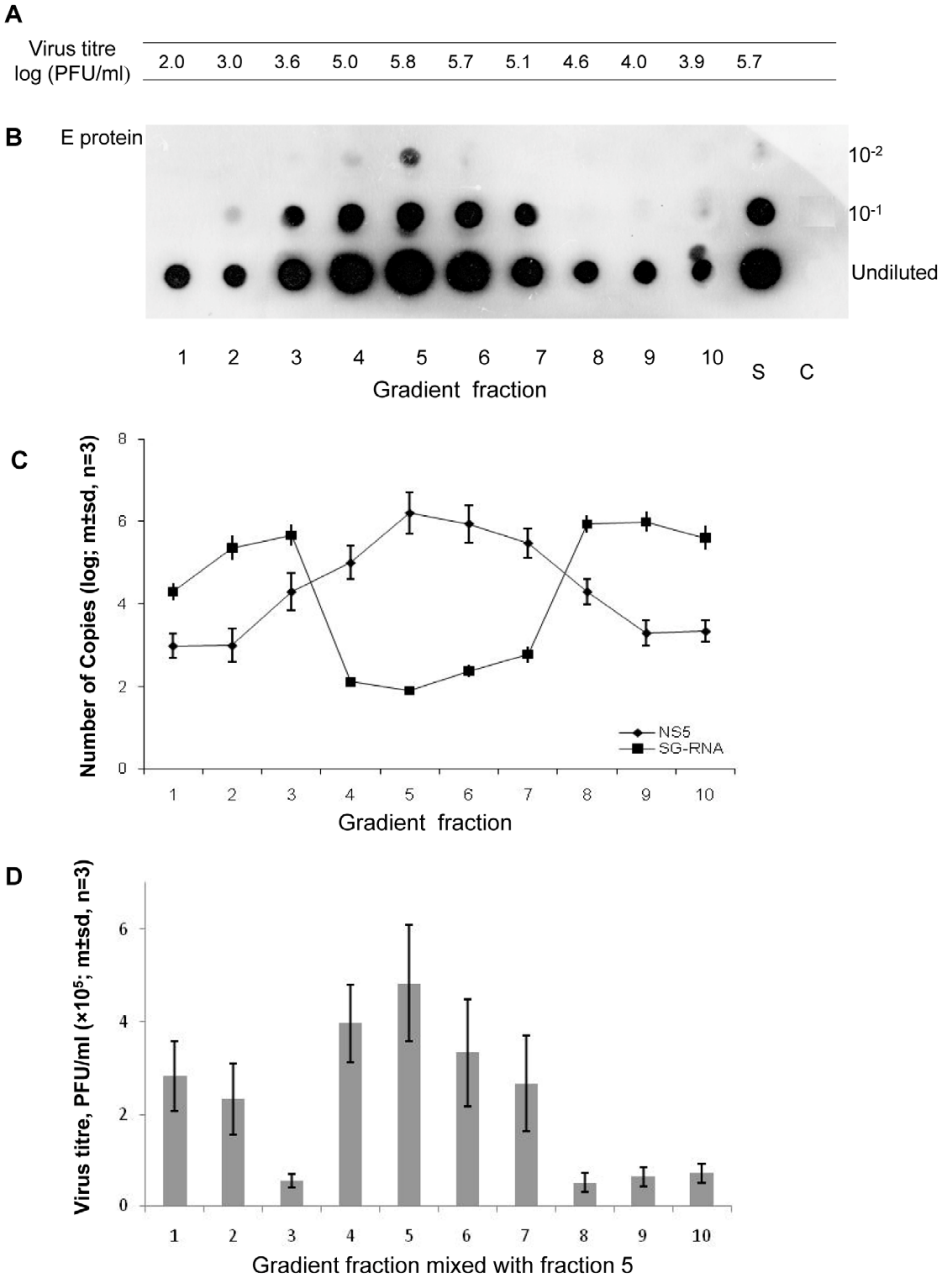
Properties of DENV 2 particles separated by centrifugation on a 5–50 per cent continuous sucrose gradient. (A) Titre of virus in each gradient fraction and in the DENV 2 stock (S) added to the gradient (fraction 1 contains 50% sucrose/PBS and fraction 10, 5% sucrose/PBS). (B) Titre of envelope protein detected by indirect ELISA on gradient fractions blotted onto nitrocellulose membrane. (C) DENV 2 NS5 and 290 nt DENV 2 RNA quantified by qPCR (D) Titre of DENV 2 in culture supernatant seven days after sucrose gradient fractions were mixed with gradient fraction 5 and used to infect cultures of C6-36 cells.

In an attempt to separate DENV 2 virions with full length genomes from particles containing sub-genomic RNA, a DENV 2 virus stock (isolate 61452) that contained sub-genomic DENV RNA was centrifuged on 5–50% w/v sucrose/PBS gradients. Ten fractions were collected (fraction 1, at the bottom of the gradient, was the most dense) and assayed for infectivity by plaque assay (Figure 6 A) and for DENV E protein by dot blotting (Figure 6B). Quantitative RT-PCR was used to quantify the 290 nt sub-genomic RNA and a region of the NS5 gene which served as a surrogate for full length DENV genomes (Figure 6 C). The highest titres of infectious virus were detected in fractions 4, 5 and 6 (Figure 6A). Although the DENV envelope (E) protein was detected in all gradient fractions, its concentration in each fraction mirrored the titres of infectious virus. Putative full length DENV 2 genomes also were most abundant in those fractions that contained most infectious virus (Figure 6 C). Most 290 nt sub-genomic RNA was detected in fractions 1, 2, 3, 8, 9 and 10, where infectious virus was less abundant. The elevated levels of sub-genomic RNA in denser fractions (1, 2 and 3 at the bottom of the gradient) which were accompanied by reduced virus titres, may reflect aggregates of viral particles containing the 290 nt sub-genomic RNA.

To determine if the gradient fractions containing the sub-genomic DENV 2 RNA interfered with the replication of homologous, infectious virus, aliquots from gradient fraction 5, which contained most infectious virus, were mixed with an equal volume of each gradient fraction and used to infect C6/36 cells. The titre of infectious virus in the supernatant from these cultures was determined 7 days post -infection using an infectious focus assay on BHK – 21 clone 15 cells. Addition of aliquots from fractions 3, 8, 9 and 10 caused a significant reduction (p<0.05; student t-test) in the yield of virus from C6-36 cells infected with DENV from gradient fraction 5 (Figure 6 D).

## Discussion

Sub-genomic DENV RNAs, representing each of the four DENV serotypes, were detected in sera from most dengue patients and from the corresponding isolates by 3′ RACE. Subsequent investigations, focussing on DENV 2, provided evidence of DEN virus-like particles that satisfy the criteria for DI particles [4]. While there is an extensive literature describing DI particles produced *in vitro* or in other experimental systems [9], this is the first report, to our knowledge, of DI particles in an acute viral infection in humans. Others have identified large deletions in the genomes of viruses like Hepatitis A, which also cause acute infections [15], but they have not satisfied the remaining criteria for DI particles. While there is an extensive literature elaborating the RNA elements required to generate negative and positive strands of flavivirus RNA and for packaging of the RNA *in vitro* or in experimental systems [23]–[28], this study points to the minimum elements required for these activities during natural cycles of transmission.

For the following reasons, we do not believe the short pieces of cDNA, which corresponded to DENV RNA with all the key elements required for its replication [23], [24] were *in vitro* artefacts. No cDNA was detected following PCR if the primer was omitted from the RT step (Figure 2 B) i.e. the cDNA fragments were not artefacts resulting from “self-priming” in PCR reactions [22]. The RT PCR reactions which generated the short cDNA fragments from RNA from sera from some dengue patients failed to generate short fragments of cDNA when a wide range of concentrations of RNA transcribed from a DENV 2 infectious clone was employed (Figure 2 A) or when RNA was recovered from infectious DENV 2 derived from C6-36 cells transfected with this RNA (Figure 3 B, passages 2 and 3). However, RT PCR employing RNA recovered after prolonged culture or repeated passage (Figure 3 A and B) of the same DENV 2 derived from this infectious clone gave rise to short pieces of cDNA almost identical to those detected when using sera from patients infected with DENV 2 (Table 1).

While it was possible to detect 290 nt sub-genomic DENV RNA in infected cells using probes corresponding to 5′ and 3′ regions of the DENV 2 genome (Figure S1), the equivocal results obtained when attempting to detect these short pieces of RNA in culture supernatant from DENV 2 infected cells using northern blots, may have been due to the limited sensitivity of our assay. Detection of negative strand RNA corresponding to this sub-genomic 290 nt RNA (Table 2) in C6-36 cells infected with a DENV 2 population which contained the corresponding piece of positive sense sub-genomic RNA, suggested that the 290 nt positive strand of RNA was being replicated or that new sub-genomic negative strand RNA was being produced or that both processes were occurring in the infected cells (see below). The ratio of positive to negative strand sub-genomic RNA was similar to that described previously for full length DENV genomes [29].

The nucleotide sequences of the DENV DI RNA usually contained a variable-length, A-rich, sequence at the crossover site that was shared by both the 5′ and 3′ elements in the full length DENV genome (Table 1). This is a hallmark of imprecise recombination by the copy-choice mechanism during RNA replication [30], in which the replication complex “jumps” from a recombination hotspot on one RNA strand to an homologous sequence on a second similar or identical RNA strand. The CS and UAR sequences of the DENV genome are believed to bring the 3′ and 5′ ends of the genome together such that it forms a “panhandle” structure [23]–[28]. The RdRP then binds to the 5′ end of the genome in order to initiate negative strand RNA synthesis at the adjacent 3′ end of the genome. Intriguingly, these features also might facilitate a novel intra-strand recombination mechanism to produce DENV DI RNA *de novo*. As circularisation of the DENV RNA genome brings the 5′ and 3′ ends into close proximity, recombination could occur in *cis* between two adjacent homologous sequences near the 5′ and 3′ ends of the genome (Table 1) during negative strand synthesis resulting in the deletion of the majority of the genome. It has been suggested that alphavirus DI particle RNA with large internal deletions may arise by sequential deletion events [31]. However, the failure to detect any DENV RNAs of between approximately 1 and 6 Kb suggests that shortening of the DENV genome does not occur in a similar stepwise manner. From the discussion above, it seems unlikely that the sub-genomic RNA detected in these DENV infections arose by a similar mechanism, or plays a similar role, as the 300–500 nucleotide sub-genomic fragments from the 3′ region of the genome of West Nile virus described elsewhere [32].

The observation that a 290 nt strand of positive sense RNA, corresponding to that detected in infected cells, could be packaged into particles if fully functional genomes were present (Figure 4) is in agreement with the previous observation that Yellow Fever virus could encapsidate RNA in *trans* [33] but contrasts with reports that Kunjin virus appears unable to do so [34], [35]. This may reflect a segregation of encapsidation processes between encephalitic and haemorrhagic members of the flavivirus genus.

When infected with DI particle-free DENV, the human cell line (HuH7, liver) produced sub-genomic RNA more rapidly than did mosquito cells (Figure 3). Alternate passage of extracellular virus in human and mosquito cells did not eliminate particles containing sub-genomic RNA. However, RNA recovered from culture supernatants during the first two passages in HuH7 cells and the first alternating passages in C6-36 and HuH7 cells gave rise to cDNA of varying sizes (Figure 3 C,D) suggesting that some short RNAs were being lost and new ones generated until an optimal form of the short RNA was reached. The 28 days required for DI particles to appear in mosquito cells is much longer than the life of most *Aedes aegypti* mosquitoes in nature (average 8.6 days [36]), while the 7 days or less required for the appearance of DI particles in human cells would be sufficient time for them to be generated and included in a blood meal taken by a mosquito from a human host [37]. It is not clear why DI particles were not detected in all patients infected with the same DENV serotype during the same outbreak (Table S 1). It is possible that the 3′ RACE protocol employed lacked the necessary sensitivity. Alternatively, some host factors may be in play which rapidly eliminate DI particles or which restrict their formation e.g. the temperature or duration of fever might influence the fidelity of the RdRP or the frequency of recombination, if that is how DENV genomes with large internal deletions are derived. Nonetheless, these observations raise the possibility that DENV DI particles are not transmitted efficiently from host to host and that they usually arise *de novo*. The biological significance of our observations may depend on which of these two processes are in play, although they are not mutually exclusive. However, the course of an infection in a human infected with a mixture of wild type and DI DENV may be different to that in someone who only begins to generate significant numbers of DI particles 5–7 days after being infected (Figure 4D). Since there appears to be a correlation between levels of viraemia in dengue patients and disease severity [37], production of DI particles which, in turn, reduced viraemia has the potential to attenuate symptoms. This is unlikely to be an all-or-none phenomenon and the data in Figures 4 and 5 suggest that there may need to be a large excess of sub-genomic to genomic DEN RNA for any significant dampening of a viraemia.

Several replicative steps might be inhibited by DENV DI particles or their genomes. The first is competition for ribosomes after release of RNA from infecting virus particles; a second may be competition with functional genomes for NS5 and associated replicative complex proteins – the sub-genomic RNA retained all elements required for its replication. Another is competition with functional genomes for capsid proteins. The second would have a direct effect on the number of full-length genomes produced and it is compatible with the observation that large numbers of sub-genomic RNAs/DI particles were needed to reduce the output of functional, infectious virus (Figures 4–6). Models of DENV RNA replication suggest that up to five nascent strands of positive sense RNA may be generated from a single Replicative Intermediate (RI) [38]. However, RIs of the sub-genomic RNAs detected in this study probably would be too short to accommodate more than one nascent strand. In these circumstances, DI particles containing sub-genomic RNA may need to be in significant excess in order to overcome the replicative advantage of long genomes able to support simultaneous production of multiple new genomes.

DENV DI particles may not be a novel aspect of DENV biology but part of a broad spectrum of defects in the viral genome which attenuate disease and make these viruses very effective parasites. Attempts to correlate DENV DI particle numbers in patients with the severity of symptoms were thwarted, in part, by a lack of sufficient subjects with severe symptoms. However, since DENV populations in patients contain large numbers of virions with defective genomes [17], [18], [39], it would have been impossible to distinguish the effects due to DI particles with genomes containing the large deletions observed in this study from those due to full length genomes with defects or due to genomes with small numbers of nucleotide insertions or deletions. We propose that DENV DI particles and virions with full length, but defective, genomes which are unable to generate infectious progeny, reduce the severity of symptoms in humans, enabling those with mild disease to remain mobile and to disseminate DENV more widely than can *Aedes* mosquitoes whose average flight range is approximately one hundred metres [40], [41]. Human mobility has been shown to be critical to the maintenance of cycles of transmission of vector-borne viral diseases [19].

## Materials and Methods

### Ethics statement

This study was approved by the Queensland University of Technology Research Ethics Unit (Ethics No. 0700000910). As no patient tissue was employed in this study, the University Ethics Unit did not require informed patient consent. All patient identifiers were removed from dengue virus samples before they were used for research purposes.

### Cells and sera

*Aedes albopictus* mosquito cells (C6/36) were maintained at 30°C, and human hepatocellular (HuH7) and BHK-21 clone 15 cells [42] at 37°C in RPMI 1640 (Gibco, USA) supplemented with 5% v/v fetal calf serum (FCS) and 100 U/ml penicillin, 100 µg/ml streptomycin and 2 mM glutamine (Gibco, USA) (growth medium).

Sera were collected from dengue patients by venepuncture (Ethics approval QUTHREC0700000910). DENV were recovered from the sera by culturing them with C6/36 cells for 7 days at 30°C. The viruses were serotyped by indirect immunofluorescence employing serotype specific monoclonal antibodies [43]. Virus isolates were stored at −80°C until required.

### 3′ rapid amplification of cDNA ends (RACE) for detection of sub-genomic DENV RNA

RNA was extracted from sera, virus isolates or from sucrose gradient fractions using QIAamp Viral RNA Mini purification kits (QIAGEN, Germany), and from DENV infected cells using NucleoSpin RNAII purification kits (Macherey-Nagel, Germany) according to the manufacturer's instructions. The 3′RACE procedure was a modification of an earlier protocol [44]. Briefly, an oligonucleotide primer (adaptor primer 1, Table S2) was ligated to the 3′ end of RNA extracted from DENV or DENV-infected cells using RNA ligase (Promega, USA) according to the manufacturer's instructions. Reverse transcription was performed using adaptor primer 2 (Table S2), the sequence of which was complementary to that of adapter primer 1, and superscript III reverse transcriptase (Invitrogen, USA) following the product protocol. In the subsequent PCR reaction, various primer combinations were used. The principal primer combination was a pair corresponding to the 5′ and 3′ ends of the DENV genome. Since the 5′ sequences of DENV 1-3 are conserved, a pair of primers (D2-5′UTR-F and D2-3′UTR-R) could be used for the samples of DENV 1-3. The primers employed for amplification of DENV-4 cDNA were D4-5′UTR-F and D4-3′UTR-R (Table S2). Other primer combinations included D2-5′UTR-F and adapter primer 2, D2-9678-F and adapter 2, and D2-8375-F and D2-8885-R. Unless indicated otherwise, the PCR reactions were carried out with one cycle at 94°C for 2 min followed by 40 cycles of 94°C for 30 sec, 56°C for 30 sec and 72°C for 2 min and completed with one cycle of 72°C for 10 min.

In order to determine the sequences of the cDNA generated, the PCR products were cloned and sequenced. An A-tail was added to both ends of PCR products using Taq polymerase (Promega, USA) and dATP. The A-tailed product was ligated into pGEM-T vectors (Promega, USA) according to manufacturer's instruction and used to transform *E. coli* DH5α. Plasmids were recovered from colonies of bacteria which grew on Luria-Bertani agar supplemented with 100 µg of amplicillin/ml, 0.5 mM isopropyl-β-D-thiogalactopyranoside (IPTG), and 80 µg of bromo-4-chloro-3-indoyl-β-D-galactosidase/ml using QIAprep Miniprep kits (Qiagen, Germany).Inserts in the plasmids were sequenced using M13 primers on an automated sequencer (ABI Prism, Australian Genome Research Facility).

Predictions of the secondary structures of sub-genomic RNAs were obtained using software available at http://mfold.rna.albany.edu/ [45].

### Quantitative real-time PCR

RNA was reverse transcribed at 50°C for 1 h using Superscript III RT (Invitrogen, USA) and random oligonucleotide primers (Roche, Switzerland). PCR was performed on a Roter-Gene-6000 real-time PCR machine (Corbett, Australia) using Light Cycler FastStart DNA Master Plus SYBR green I (Roche, Switzerland) according to the manufacturer's instructions. Cycling conditions were 1 cycle of 95°C for 10 min followed by 40 cycles of 95°C for 10 sec, 58°C for 10 sec and 72°C for 30 sec. DENV-2 sub-genomic RNA was quantified using primers D2-5′UTR-F and D2-3′UTR-R (Table S2) and a region of the NS5 gene quantified using primers D2-8375F and D2-8885R (Table S2), as a surrogate for a full length genome.

### Strand-specific RT-PCR

Positive and negative sense RNA that was used as controls in the strand-specific RT-PCR was transcribed from pGEM-D2-DI plasmid (see below) using T7 and sp6 polymerases respectively. In RT reactions, various amounts of positive and negative strand RNA was mixed with primer D2-3′UTR-R (for positive strand detection) or with primer D2-5′UTR-F (for negative strand detection) and denatured at 90°C for 3 min, RT reactions were carried out at 65°C for 1 h in the presence of Thermscript Reverse Transcriptase (Invitrogen, USA) followed by heating to 85°C for 10 min to inactivate the RT. The RT products then were treated with 200 U/ml RNase H and 1000 U/ml RNase T1 for 10 min at 37°C. The qPCR was performed on a Roter-Gene-6000 real-time PCR machine (Corbett, Australia) using Light Cycler FastStart DNA Master Plus SYBR green I (Roche, Switzerland) and primers D2-5′UTR-F and D2-3′UTR-R (Table S2). Cycling conditions were 1 cycle of 95°C for 10 min followed by 35 cycles of 95°C for 10 sec, 58°C for 10 sec and 72°C for 30 sec.

### Transcription of RNA from plasmids and transfection of C6-36 cells

Plasmid pWSK601, coding for the genome of DENV 2 [46], was a gift from Dr Andrew Davidson, Monash University Australia. Plasmids pGEM-D2-5′-206 and pGEM-D2-3′-10526 were constructed by inserting PCR fragments corresponding to DENV2 nt 1-206 or nt 10526-10723 into a pGEM-T vector. These fragments were derived from plasmid pSWK601 by PCR employing primers D2-5′UTR-F and D2-206-R (pGEM-D2-5′-206) and primers D2-10526-F and D2-3′UTR-R (pGEM-D2-3′-10526). Plasmid pGEM-D2-DI was pGEM-T with a 290 nt cDNA insert corresponding to the 290 nt DENV 2 sub-genomic RNA detected in serum from a patient with a DENV 2 infection i.e. nt 1-190 from the 5′ end of the genome linked to nt 10526-10723 from the 3′ end of the genome. pUC-D2-DI was constructed by inserting a T7 promoter sequence and the 290 nt DENV 2 sub-genomic ds-cDNA into a pUC18 vetor. A control plasmid, pUC-DENV-5′ contained the T7 promoter sequence, cDNA corresponding to the 5′ 373 nucleotides from DENV 1 and a D2-3′XbaI-R primer sequence (Table S2).

Plasmids were linearized by treatment with the corresponding restriction enzyme followed by column purification (Roche). Transcription was performed using the MEGAscript transcription system (Ambion, USA) according to the manufacturer's instruction.

C6/36 cells were transfected by electroporating 10^7^ cells in 0.5 ml PBS with 10 µg of RNA, transcribed from one of the plasmids above, in a Gene Pulser II (Bio-Rad, USA) at 550 µF and 250 V. After electroporation, the cells were diluted in fresh growth medium and transferred to a 25 cm^2^ tissue culture flask at 30°C. The next day, the growth medium was replaced with fresh medium and the cells incubated for a further 6 days. Supernatant from this culture was used to infect a fresh flask of C6/36 cells in order to prepare working stocks of virus.

### Northern Blot

Plasmids pGEM-D2-5′-206 and pGEM-D2-3′-10526 were linearized using restriction enzyme Sac II followed by purification with a PCR purification Kit (Roche, Germany). DIG-labelled negative strand RNA probes were synthesised from this linearised DNA using sp6 polymerase from a DIG Northern Starter Kit (Roche) according to the manufactures instructions. Briefly, RNA was denatured in loading buffer for 10 min at 65°C and separated in 1.2% formaldehyde agarose gels followed by capillary transfer onto positive charged nylon membrane (Roche, Germany) and UV crosslinking with 250 nm UV light for 2.5 min. The membrane was prehybridized in hyb granules buffer for 30 min and then hybridized with probe for 6 h at 68°C. After stringent washes, the membrane was blocked with block buffer from the kit followed by incubation with alkaline phosphatase-labelled anti-DIG antibody for 30 min at RT. The membrane then was washed twice in wash buffer and incubated in CDP-Star agent at RT for 5 min before being exposed to photographic film (Agfa, Belgium).

### Sucrose gradient centrifugation

Two ml of supernatant from cultures of C6/36 cells infected with DENV-2 (D2Myanmar08.61452), in which a 290 nt sub-genomic DENV RNA had been identified, was loaded on a 10 ml 5–50% w/v sucrose/PBS (pH 7.4) gradient and centrifuged at 25,000 r.p.m. for 2.5 h at 4°C in an SW40 rotor (Beckman, USA). Fractions (1 ml) were collected from the gradient by puncturing the bottom of the centrifuge tube.

### Dot blotting

Supernatant from cultures of DENV 2 infected C6-36 cells or fractions from sucrose gradients were diluted 10-fold in PBS and 100 µl aliquots were added to nitrocellulose membrane (Amersham Biosciences, Sweden). The membrane then was blocked in 4% w/v skim milk (Diploma, New Zealand) and 1% v/v FCS in PBS at 4°C overnight before being incubated in HRP-labelled anti-flavivirus antibody (6B6C.1, Panbio, Australia) in blocking buffer for 1 h at room temperature. After four washes in PBS, the nitrocellulose was incubated with lumi-light western blotting substrate (Roche, Germany) for 5 min and then exposed to photographic film (Agfa, Belgium).

### Infectious focus assay

Ten fold dilutions of virus in culture medium supplemented with 2% v/v FCS were added to monolayers of BHK-21 clone 15 cells in 24 well plates (Thermo Fisher Scientific, Denmark), incubated at 37°C for 1 h and then overlayed with 1 ml of 1.5% w/v carboxymethylcellulose (BDH, UK) in culture medium containing 2% v/v FCS. After 4 days at 37°C, the CMC overlay was removed and the cells were fixed with 5% v/v formaldehyde/PBS for 30 min followed by 5 min incubation in 5% v/v Triton-X-100/PBS. After discarding the Triton-X-100 and washing twice with PBS, 2% w/v skim milk/PBS was added for 30 min at room temperature. The blocking buffer then was replaced with HRP-6B6C.1 antibody diluted in blocking buffer and incubated for 1 h at room temperature. After 4 washes with PBS, 200 µl of insoluble tetramethylbenzidine solution (Sigma, USA) was added to the cell monolayers and incubated at room temperature until well defined foci were visible.

## Supporting Information

Figure S1 **Northern blot analysis of RNA from DENV 2 isolate (61452) and from C6-36 cells infected with this virus.** (A) Blots probed with a DIG-labelled probe complementary to the DENV2 5′1-206 nt (5′-probe). Gen., genome length RNA transcribed from DENV 2 infectious clone pWSK601; SG, 290 nt sub-genomic DENV 2 RNA transcribed from plasmid pGEM-D2-DI; UC, cellular RNA from uninfected C6-36 cells; Cellular RNA, from cultures of C6-36 cells infected with DENV 2 [61452] 5 or 8 days previously; RNA from Supernatant, RNA recovered from the supernatant of the cultures of C6-36 cells infected with DENV 2 [61452] 5 or 8 days previously. (B) As for (A) but analysed with a DIG-labelled oligonucleotide probe complimentary to the 3′10526-10723 nt of DENV 2 (3′-probe).
(TIF)

Table S1 **Sub-genomic RNA detected in sera from dengue patients.**
(DOC)

Table S2 **Sequences of oligonucleotide primers employed in this study.**
(DOC)
