# Supplementary figures and images for: Defective Interfering Viral Particles in Acute Dengue Infections
Source: PLoS One. 2011 Apr 29;6(4):e19447. doi: 10.1371/journal.pone.0019447 (PMC3084866; doi:10.1371/journal.pone.0019447)

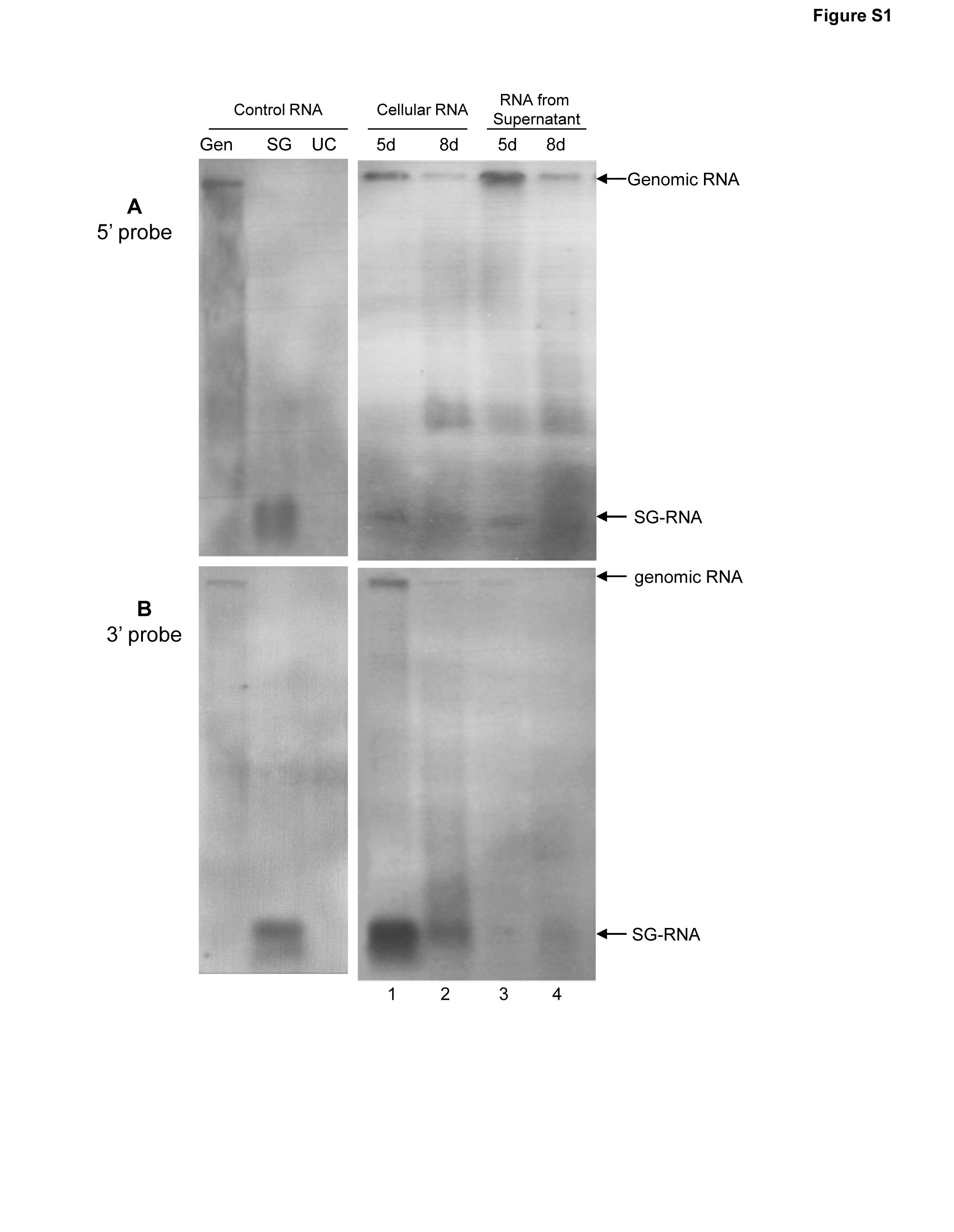

Supplement: Figure S1 — Northern blot analysis of RNA from DENV 2 isolate (61452) and from C6-36 cells infected with this virus. (A) Blots probed with a DIG-labelled probe complementary to the DENV2 5′1-206 nt (5′-probe). Gen., genome length RNA transcribed from DENV 2 infectious clone pWSK601; SG, 290 nt sub-genomic DENV 2 RNA transcribed from plasmid pGEM-D2-DI; UC, cellular RNA from uninfected C6-36 cells; Cellular RNA, from cultures of C6-36 cells infected with DENV 2 [61452] 5 or 8 days previously; RNA from Supernatant, RNA recovered from the supernatant of the cultures of C6-36 cells infected with DENV 2 [61452] 5 or 8 days previously. (B) As for (A) but analysed with a DIG-labelled oligonucleotide probe complimentary to the 3′10526-10723 nt of DENV 2 (3′-probe). (TIF) [file pone.0019447.s001.tif]
