# Supplementary material for: Defective Interfering Viral Particles in Acute Dengue Infections
Source: PLoS One. 2011 Apr 29;6(4):e19447. doi: 10.1371/journal.pone.0019447 (PMC3084866; doi:10.1371/journal.pone.0019447)
Supplement: Table S1 — Sub-genomic RNA detected in sera from dengue patients. (DOC) [file pone.0019447.s002.doc]

**Table S1. Sub-genomic** RNA detected in sera from dengue patients.

| Serotype | Size of cDNA (bp) | Samples with cDNA of size shown/Samples tested |
| --- | --- | --- |
| DENV1 | 370 | 3/8 |
|  | 395 | 1/8 |
|  | No cDNA | 4/8 |
| DENV2 | 290 | 1/2 |
|  | 400 | 2/2 |
|  | No cDNA | 0/2 |
| DENV3 | 390 | 2/3 |
|  | No cDNA | 1/3 |
| DENV4 | 510 | 2/9 |
|  | 640 | 6/9 |
|  | 840 | 2/9 |
|  | 1030 | 4/9 |
|  | No cDNA | 0/9 |
