# Supplementary material for: Defective Interfering Viral Particles in Acute Dengue Infections
Source: PLoS One. 2011 Apr 29;6(4):e19447. doi: 10.1371/journal.pone.0019447 (PMC3084866; doi:10.1371/journal.pone.0019447)
Supplement: Table S2 — Sequences of oligonucleotide primers employed in this study. (DOC) [file pone.0019447.s003.doc]

**Table S2. Sequences of oligonucleotide primers employed in this study.**

| Primer name | Primer sequence |
| --- | --- |
| Adaptor primer1 | 5’ AATTCCCGGGTCAGAGCTGTTTCCTGGGATCCTGCA  GAATTCT 3’ |
| Adaptor primer2 | 5’ GGATCCCAGGAAACAGCTCTGAC 3’ |
| D2-5’UTR-F | 5’AGTTGTTAGTCTACGTGGACC 3’ |
| D2-3’UTR-R | 5’ AGAACCTGTTGATTCAACGGC 3’ |
| D4-5’UTR-F | 5’ AGTTGTTAGTCTGTGTGGACC 3’ |
| D4-3’UTR-R | 5’ AGAACCTGTTGGATCAACAAC 3’ |
| D2-8375-F | 5’ GCTCTCAGTGAACTGCCGGAGACCC 3’ |
| D2-8885-R | 5’ TCCCAAGACCCATTAACACTGTGGC 3’ |
| T7-D2-5’-F | 5’ GCGGCCGCGTAATACGACTCACTATAGGAGTTG  TTAGTCTACGTGGACCG 3’ |
| D2-206-R | 5’ CATTCCAAGTGAGAATCTCTTTGTC 3’ |
| D2-10526-F | 5’ AATCGCAGCAACAATGGGGGCCC 3’ |
| D2-3’/XbaI-R | 5’ ATCTAGAAGAACCTGTTGATTCAACAGCAC 3’ |
